# Preoperative Neck Angulation is Associated with Aneurysm Sac Growth Due to Persistent Type Ia Endoleak after Endovascular Abdominal Aortic Aneurysm Repair

**DOI:** 10.3400/avd.oa.20-00057

**Published:** 2020-09-25

**Authors:** Yoshimasa Seike, Tetsuya Fukuda, Koki Yokawa, Yosuke Inoue, Takayuki Shijo, Kyokun Uehara, Hiroaki Sasaki, Hitoshi Matsuda

**Affiliations:** 1 Department of Cardiovascular Surgery, National Cerebral and Cardiovascular Center; 2 Department of Radiology, National Cerebral and Cardiovascular Center

**Keywords:** endovascular aneurysm repair, instructions for use, angulation, endoleak

## Abstract

**Objective**: This study aims to determine how instructions for use affect the occurrence of aneurysm sac growth and endoleaks after an endovascular aneurysm repair (EVAR).

**Materials and Methods**: We reviewed 302 patients who underwent EVAR for abdominal aortic aneurysm between 2007 and 2013, and we were able to enroll 159 patients (74% men, mean age 78±7 years) with adequate data (mean follow-up; 48±20 months).

**Results**: The angle of the proximal landing zone (LZ) (hazard ratio: 1.02, 95% confidence interval: 1.00–1.03, p=0.01) was recognized as an independent risk factor of sac growth (≥5 mm). The receiver operating characteristics curve (area under the curve: 0.72) showed a cutoff value of 47° of the minimum angle of the proximal LZ to predict sac growth. Freedom rates for persistent type Ia endoleaks were also found to be lower in the angulated group than those in the other groups (p=0.0095, log-rank).

**Conclusion**: The angle of the proximal LZ was identified as an independent risk factor for sac growth post-EVAR. The incidence of persistent type Ia endoleaks was significantly higher in the angulated group.

## Introduction

Endovascular aneurysm repair (EVAR) has been increasingly used for infrarenal abdominal aortic aneurysm (AAA) repair, mainly due to its lower short-term morbidity and mortality compared with open surgery in the elderly. Nonetheless, a major disadvantage of this technique is the possibility for secondary intervention due to any type of endoleaks.^1–3)^

Recently, various stent grafts have become commercially available, and each device has its own instructions for use (IFU), which indicate the anatomic restrictions for the device selection to minimize adverse outcomes after EVAR. These anatomic restrictions mainly consist of the aortic neck diameter, aortic neck length, aortic neck angulation, and the parameters of the iliac arteries. However, many elderly patients necessitate and undergo EVAR even if the anatomy of their AAA is beyond the IFU parameters. Besides its clinical importance, IFU adherence remains controversial as that is said by the reported studies.^4–6)^

This study aimed to determine how IFU influences the incidence of sac growth and endoleaks post-EVAR and to recognize the computed tomography (CT) features of IFU, which may predict adverse events post-EVAR.

## Materials and Methods

### Ethics statement

This observational study was approved by the Institutional Review Board (M30-036) at our center, and individual oral and written informed consents were waived because of its retrospective nature.

### Study population

**Figure 1:**
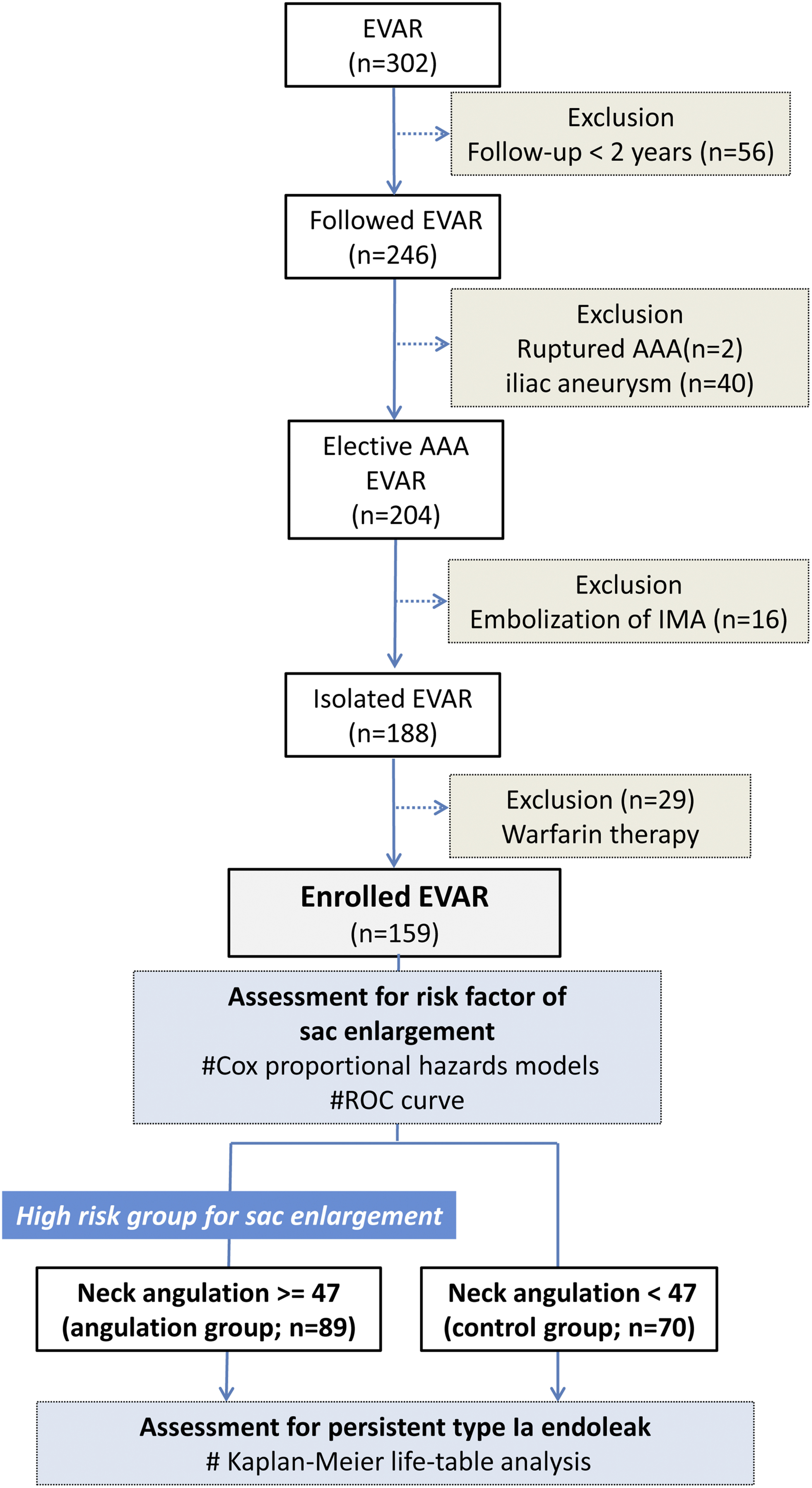
Fig. 1 Flowchart of study population and method.

The data were collected from hospital admission and outpatient medical records. All patients have been followed up as outpatients either at our center or by a local physician. The medical records of 302 patients who underwent EVAR between January 2007 and December 2013 were retrospectively reviewed. More contemporary cases were not studied, because we have performed intraoperative aneurysm neck and sac embolization with N-butyl-2-cyanoacrylate (NBCA) to treat type Ia endoleaks during EVAR since 2014, besides using several other techniques including inferior mesenteric artery (IMA) embolization using coil devices. The patients who received CT scans for follow-up medically over 2 years after EVAR and had undergone elective EVAR for infrarenal AAA were included. Forty patients (14.1%) with a solitary iliac aneurysm and two patients (0.7%) with a ruptured AAA were excluded from the study. In addition, 16 patients (5.6%) with IMA embolization and 29 patients (1.0%) who had received warfarin therapy were also excluded from the study; as what we had previously reported, warfarin therapy was considered a substantial risk for sac enlargement after EVAR.^7)^ The patients with type I and type II endoleaks were included, while patients with type III and type IV endoleaks were excluded from the study. Finally, follow-up data were available in 159 patients (74% men, mean age 78±7 years, mean follow-up time 48±20 months) (**Fig. 1**).

### Device for EVAR

Stent graft selection depends on factors such as its availability, anatomical suitability, and the surgeon’s preference. EVAR was done using Excluder (W. L. Gore and Associates, Flagstaff, AZ, USA) in 69 patients (43.4%), Zenith (COOK Medical, Bloomington, IN, USA) in 44 (27.7%), Endurant (Medtronic Cardiovascular, Santa Rosa, CA, USA) in 28 (17.6%), Powerlink (Endologix, Irvine, CA, USA) in 17 (10.7%), and Talent Abdominal (Medtronic AVE, Santa Rosa, CA, USA) in 1 (0.6%).

### Measurement of CT scans

Axial CT images with a slice thickness of 2 mm were used to measure the CT scan findings. Data were transferred to a three-dimensional (3D) workstation (Ziostation2 version 2.9.2.0a; Ziosoft, Inc., Tokyo, Japan), and the size of the aneurysm sac was measured using the major and minor axes. Aneurysm morphology including suprarenal angulation, neck diameter, neck length, neck angle, and maximum sac diameter was measured by the first author and one radiologist. Reverse-tapered shape was defined as the size discrepancy more than 3 mm within 15 mm below the lower renal artery orifice.

**Figure 2:**
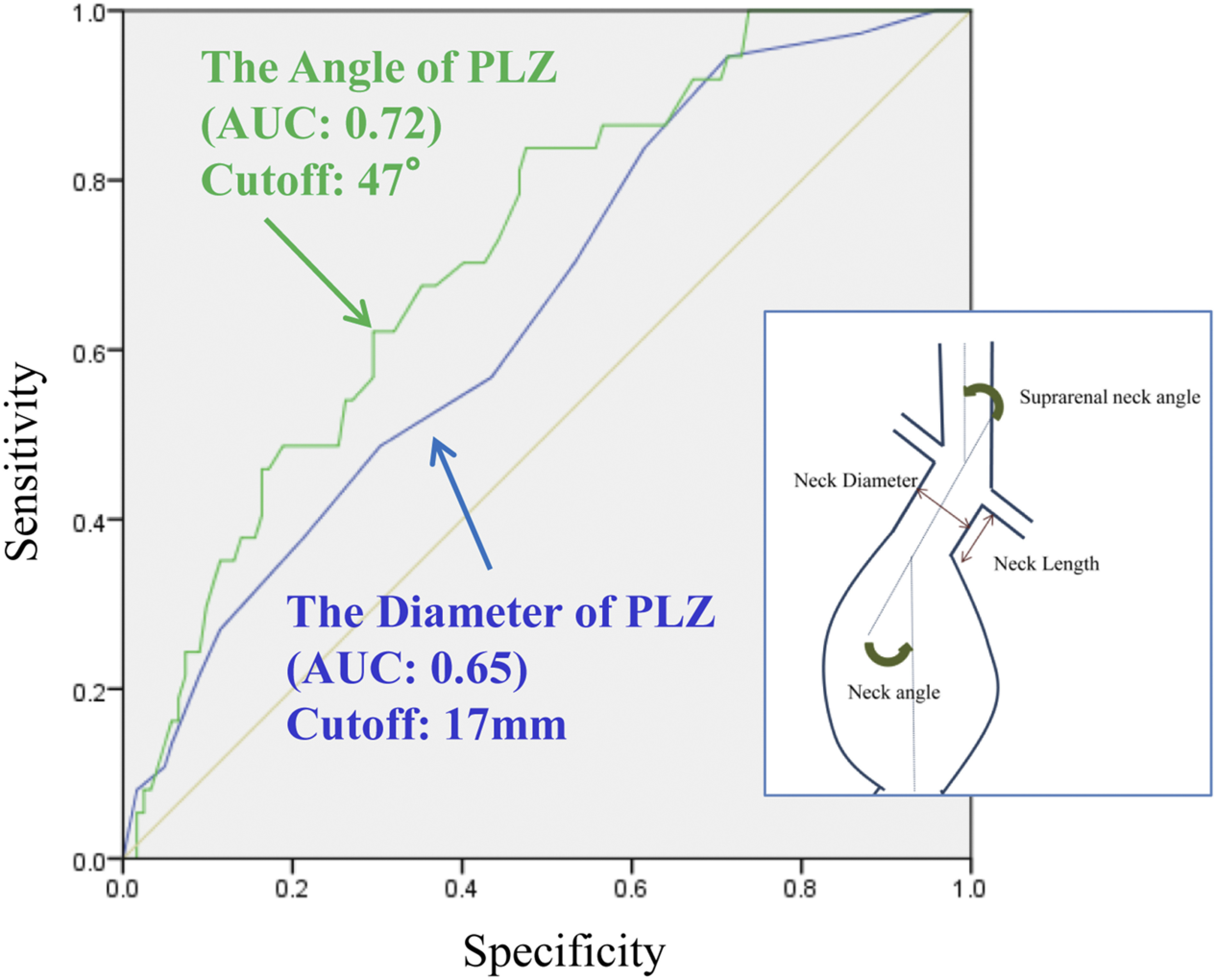
Fig. 2 Receiver operating characteristics curve analysis for angle and diameter of proximal landing zone.

Neck angle has been defined as the angle between the axes of aneurysm and the neck of aneurysm. **Figure 2** indicates the measurement of the infrarenal neck angle is the same as the neck angle. Suprarenal neck angle was defined as the angle between the axes of the suprarenal aorta and the neck of aneurysm.

During the follow-up periods, a change on the minor axis over 5 mm was considered significant for sac growth post-EVAR.^8)^ The CT scans with intravenous contrast were performed in all patients at discharge and at least twice over 2 years post-EVAR. If aneurysm sac growth was detected on CT scans postoperatively, CT angiography was performed to identify the sources of endoleaks. Persistent endoleak was defined as the endoleak which did not disappear 1 year post-EVAR, in addition to a newly developed endoleak during the follow-up period.^7)^

### Risk factors for sac enlargement

Preoperative CT findings associated with IFU are presented in **Table 1** and were evaluated using univariate analysis to find the risk factors for aneurysm sac growth post-EVAR. The receiver operating characteristics (ROC) curve was performed to evaluate the relation between the high-risk group for sac enlargement and preoperative CT findings including the angle of the proximal LZ and diameter of the proximal LZ on the occurrence of sac enlargement (**Fig. 2**).

**Table 1:** Table 1 Preoperative computed tomography findings related to instruction for use and Cox regression analysis: predictors of sac enlargement (>5 mm)

Covariate		Overall	HR	95% CI	p-value	HR	95% CI	p-value
			Univariate			Multivariate		
AAA size	(mm)	48±6	1.07	1.01–1.13	0.020	1.06	0.99–1.22	0.057
Suprarenal angulation	Angle (degree)	29±23	1.01	1.00–1.03	0.057			
Proximal LZ	Length (mm)	32±12	0.98	0.95–1.01	0.088			
	Diameter (mm)	19±3	1.14	1.04–1.26	0.008	1.11	1.00–1.24	0.042
	Angle (degree)	50±25	1.02	1.01–1.04	0.001	1.02	1.00–1.03	0.010
	Calcification (n, %)	26/159 (16%)	1.24	0.48–3.19	0.66			
	Mural thrombus (n, %)	5/159 (3.1%)	2.58	0.35–19.4	0.36			
	Reverse tapered (n, %)	8/159 (5.0%)	2.77	0.96–7.92	0.057			
	Conical (n, %)	3/159 (1.9%)	0.05	0.00–8.93	0.54			
Terminal aorta	Diameter (mm)	23±6	1.01	0.96–1.06	0.84			
Distal LZ	Length (mm)	35±12	1.01	0.98–1.03	0.82			
	Diameter (mm)	15±5	1.04	0.98–1.11	0.21			
Access route	Diameter (mm)	7.6±1.1	1.50	1.10–2.03	0.01	1.32	0.95–1.83	0.100
Embolization of bilateral IIAs	(n, %)	0/159 (0%)	—	—	—	—	—	—

AAA: abdominal aortic aneurysm; CI: confidence interval; HR: hazard ratio; IIA: internal iliac artery; LZ: landing zone

### Endpoint

To identify how IFU affects the occurrence of sac growth more than 2 years post-EVAR, risk factors related to the preoperative CT findings were examined. The patients were then divided into two groups by the cutoff value of the proximal neck angulation of 47°, and freedom from type Ia endoleak was compared with the secondary outcome indicated above (**Fig. 1**).

### Statistical analysis

All statistical analyses were performed using the SPSS software (SPSS Inc., Chicago, IL, USA). Categorical data were compared using Fisher’s exact test, while the continuous variables were expressed as mean±standard deviation and compared using a Student’s t-test. A P-value of <0.05 was considered statistically significant for all tests. Univariate and multivariate analyses were performed using the Cox hazards regression models for evaluating the time-to-event effects of the devices for EVAR and the preoperative CT findings related to IFU, including the aneurysm size, the length of the proximal LZ, the diameter of the proximal LZ, the angle of the proximal LZ, calcification, terminal aorta diameter, the length of the distal LZ, the diameter of the distal LZ, access route diameter, and embolization of the internal iliac artery. In addition, univariate analysis was performed to evaluate the effects of both immediate type Ia and type II endoleaks on sac enlargement and persistent type Ia endoleak during the follow-up period. Clinically relevant variables with a p-value <0.05 in the univariate analysis were included in the multivariate regression analyses as candidates for backward stepwise variable selections.

The probabilities predicted by ROC curve of the model were plotted, and the area under the curve (AUC) was utilized to assess the differentiation of the angle and the diameter of the proximal LZ with sac enlargement (≥5 mm). Kaplan–Meier survival curves were built to evaluate persistent type Ia endoleak, and the log-rank test was applied to compare between the subgroups (bottom row of **Fig. 1**).

## Results

### Operative results

To control an intraoperative type Ia endoleak, the adjunctive procedures were performed in ten patients using a proximal aortic cuff placement (W. L. Gore and Associates, Flagstaff, AZ, USA) and in two using a Palmaz XL stent (Cordis, Miami Lakes, FL, USA). The total number of intraoperative adjunctive procedures was 12 (7.5%). Residual intraoperative type Ia endoleak was detected in eight (5.0%) patients with minor grade. Like other endoleaks, intraoperative completion angiogram showed 45 endoleaks in 53 patients (33%), of these 44 are type II, two type III, and seven type IV.

### Sac growth

Sac growth (≥5 mm) post-EVAR was identified in 37 patients (23%). Using the Cox regression analysis, the diameter of the proximal LZ (hazard ratio [HR]: 1.11, 95% confidence interval [CI]: 1.00–1.24, p=0.042) and angle of the proximal LZ (HR: 1.02, 95% CI: 1.00–1.03, p=0.010) were identified as an independent risk factor of sac growth post-EVAR (**Table 1**). The length of the proximal LZ was also not identified as a predictor of sac growth (HR: 0.98, 95% CI: 0.95–1.01, p=0.088). As with other variables, no major devices were also identified as a predictor of sac growth (Excluder, HR: 1.30, p=0.43; Zenith, HR: 0.80, p=0.55; Endurant, HR: 0.67, p=0.41; Powerlink, HR: 0.22, p=0.14).

### ROC curves for the angle and diameter of the proximal landing zone

The ROC curve for the angle of the proximal LZ showed that AUC for the predicted probabilities was 0.72 (95% CI: 0.63–0.81). At a cutoff value of 47°, the sensitivity of the minimum angle of the proximal LZ for predicting sac growth was 83% with 51% specificity (**Fig. 2**). The ROC curve for the diameter of the proximal LZ showed the AUC of 0.65 (95% CI: 0.48–0.69), having a cutoff number of 17 mm. Pursuant to this result, the angle of the proximal LZ was detected as a significant predictor for sac growth but not its diameter.

### Presence of endoleak during the follow-up period

Postoperative CT at 1 week showed 45 endoleaks in 44 patients: 3 type Ia and 30 type II endoleaks in the angulated group and 12 type II endoleaks in the control group. Immediate type II endoleaks were more frequent in the angulated group (p=0.020) (**Table 2**). Using the univariate analysis, both immediate type Ia (HR: 3.89, 95% CI: 0.52–29.3, p=0.19) and type II (HR: 1.68, 95% CI: 0.87–3.26, p=0.12) endoleaks were found to be not a positive predictor of sac growth (≥5 mm) post-EVAR.

**Table 2:** Table 2 Patient characteristics and clinical features

Variable		Control group Angle <47 (n=70)	Angulated group Angle >47 (n=89)	p-value
Age	(years)	76±8	79±5	0.019
Female	n (%)	11 (16%)	30 (34%)	0.010
Aneurysm size	(mm)	47±6	49±6	0.002
IFU-related features				
Suprarenal angulation	Angle (degree)	16±18	37±25	<0.001
Proximal landing zone	Length (mm)	35±11	30±12	0.020
	Diameter (mm)	19±3	20±3	0.320
	Calcification (n, %)	11 (16%)	15 (17%)	1.000
	Mural thrombus (n, %)	2 (2.9%)	3 (3.4%)	0.809
	Reverse taper (n, %)	3 (4.3%)	7 (7.9%)	0.675
	Taper (n, %)	1 (1.4%)	2 (2.2%)	0.316
Terminal aorta	Diameter (mm)	21±6	24±7	0.022
Distal landing zone	Length (mm)	34±11	36±13	0.480
	Diameter (mm)	14±4	16±6	0.060
Access route	Diameter (mm)	7.4±1.0	7.6±1.2	0.300
Device-related features				
Device body diameter	(mm)	26±3.0	26±3.0	0.990
Zenith	(n, %)	19 (27%)	25 (28%)	0.890
Excluder	(n, %)	23 (33%)	46 (52%)	0.017
Powerlink	(n, %)	13 (19%)	4 (4.5%)	0.004
Talent AAA	(n, %)	0	1 (1.1%)	0.370
ENDURANT	(n, %)	15 (21%)	13 (15%)	0.260
Intraoperative features				
Intraoperative blood transfusion	(n, %)	7 (10%)	18 (20%)	0.085
Endoleak at hospital discharge				
Immediate type Ia endoleak	(n, %)	0 (0%)	3 (3.4%)	0.260
Immediate type II endoleak	(n, %)	12 (17%)	30 (34%)	0.020

AAA: abdominal aortic aneurysm; IFU: instructions for use

Two years after EVAR, 42 postoperative persistent endoleaks were detected, including eight type Ia, two type Ib, and 22 type II endoleaks in the angulation group (n=32) and one type Ib endoleak and nine type II endoleaks in the control group (n=10). In eight patients who were identified with complicated persistent type Ia endoleaks, the median proximal neck angle was determined at 73° (range, 50–116). The devices used were Excluder in five patients, Zenith in two, and Talent Abdominal in one. Median onset of endoleak confirmed by CT was determined to be at 18 months (range, 1–48). To treat sac enlargement in these patients, graft replacement was performed in two patients at 36 and 48 months post-EVAR, re-EVAR using a Gore Excluder aortic cuff (W. L. Gore and Associates, Flagstaff, AZ, USA) in two, and reinforcement with Palmaz stent (Cordis, Miami Lakes, FL, USA) in two.

### Relation between the neck angulation and persistent type Ia endoleak

As per the result of the previous ROC curve analysis, to evaluate the relation between neck angulation and persistent type Ia endoleak, the entire cohort was divided into two groups depending on the angle of the proximal LZ: 89 patients with 47° or more of the angle of the proximal LZ (angulated group, mean age 79±5 years) and 70 patients with 47° of the angle of the proximal LZ (control group, mean age 76±8 years) (bottom row of **Fig. 1**).

The angulated group was determined to be older (p=0.019), and they were mostly female compared with the control group (34% vs. 16%, respectively; p=0.043). In the angulated group, the length of the proximal LZ (35±11 mm vs. 30±12 mm, p=0.020) was determined to be shorter, while the diameter of the terminal aorta (21±6 mm vs. 24±7 mm, p=0.022) was larger. Other variables revealed no differences between the two groups (**Table 2**). Point of difference was detected in terms of the type of endograft used, including the Excluder device and the Powerlink device.

**Figure 3:**
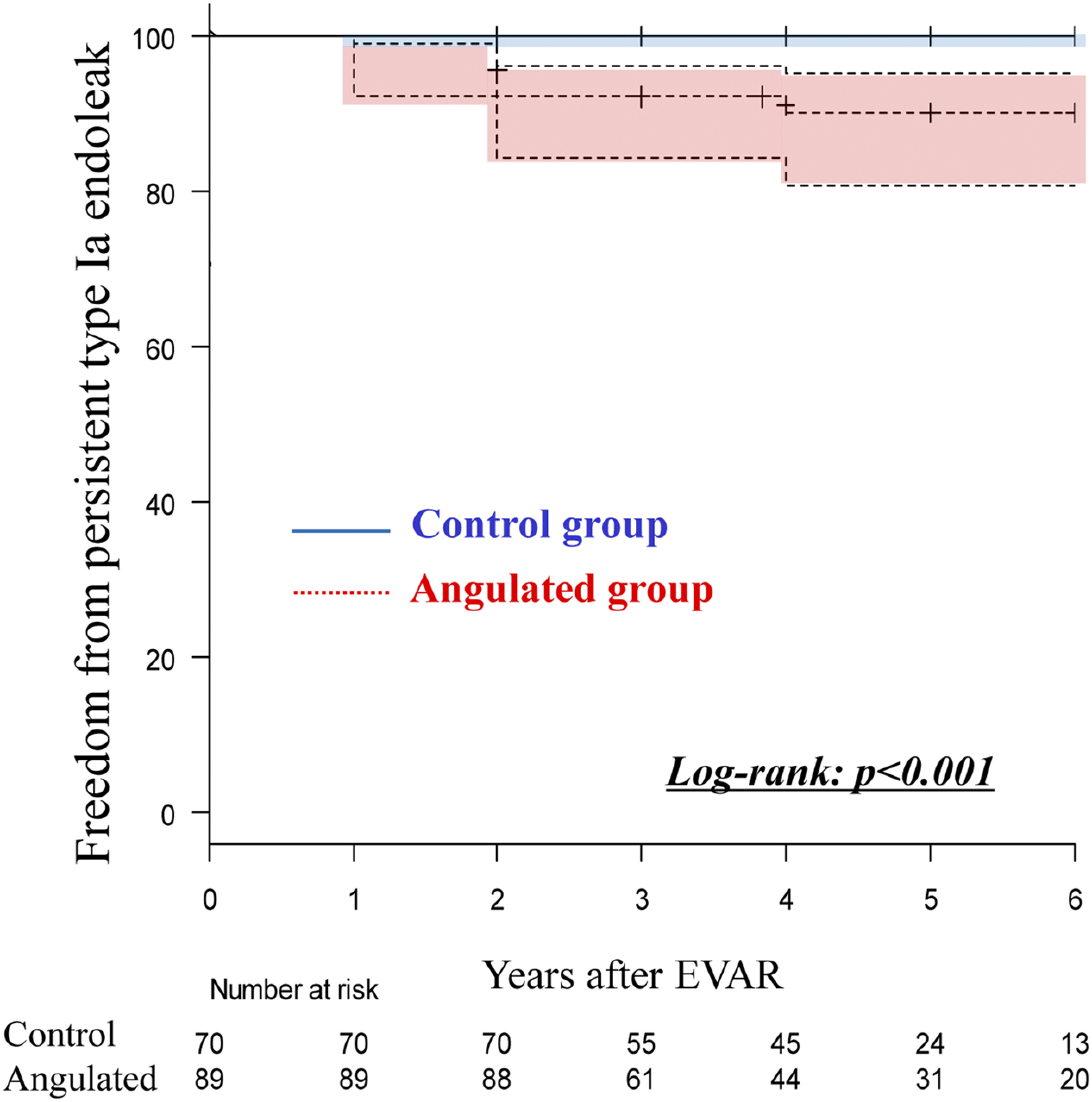
Fig. 3 The probability of freedom from persistent type Ia endoleak.

Freedom from persistent type Ia endoleak post-EVAR was determined to be higher in the angulated group compared with the control group (p=0.0095, log-rank) (**Fig. 3**). Using the Cox regression analysis, the angle of proximal LZ (HR: 1.04, 95% CI: 1.01–1.07, p=0.012) and reverse-tapered shape (HR: 6.62, 95% CI: 1.21–32.4, p=0.029) were identified as independent risk factors of persistent type Ia endoleak post-EVAR. The length of the proximal LZ was not identified as well as a predictor of persistent type Ia endoleak (HR: 0.95, 95% CI: 0.89–1.02, p=0.18) (**Table S1**). In other variables, no major devices were identified as a predictor of persistent type Ia endoleak (Excluder, HR: 2.11, p=0.31; Zenith, HR: 0.75, p=0.73; Endurant, HR: 0.04, p=0.48; Powerlink, HR: 0.04, p=0.51).

### Subgroup analysis of the angulated cases between within the IFU and outside the IFU

The angulated group (89 patients, 56%) was subsequently divided into two groups in terms of the angle of the IFU (60°) as the subgroup analysis. Thirty-six patients (36/89, 40% of angulated group) were assigned to the angulation within the IFU group (47°≤proximal neck angle <60°), and the other 53 patients (53/89, 60% of angulated group) were assigned to the angulation outside the IFU group (proximal neck angle ≥60°). Subgroups showed similar neck length (31±12 mm in the angulation within the IFU group vs. 30±12 mm in the angulation outside the IFU group, p=0.51) and neck diameter (20±3.3 mm in the angulation within the IFU group vs. 19±3.0 mm in the angulation outside the IFU group, p=0.60). Sac growth (≥5 mm) post-EVAR was observed in 11 patients (31%) of the angulation within the IFU group and in 20 patients (38%) of the angulation outside the IFU group. Meanwhile, persistent type Ia endoleak post-EVAR was observed in five patients (14%) of the angulation within the IFU group and in 20 patients (9.4%) of the angulation outside the IFU group. Freedom from sac growth (≥5 mm) was similar between the two groups (p=0.750, log-rank).

## Discussion

In 2007, a EUROSTAR study demonstrated that severe infrarenal aortic neck angulation was associated with type Ia endoleak (odds ratio 2.32, 95% CI: 1.60–3.37, p<0.0001) as the risk of the AAA outside the IFU^9)^ was related to the need for secondary interventions (HR: 1.29, 95% CI: 1.00–1.67, p=0.049). However, recently, it has been reported that there is no difference in the outcomes of EVAR between the patients within and outside of the IFU.^4–6,10,11)^

Beckerman et al. concluded that there was no difference in the all-cause mortality or the aneurysm-related mortality despite most EVAR patients being treated outside the IFU.^6)^ Moreover, aneurysm sac enlargement was identified in 11.7% of overall patients, but no significant difference between patients treated within and outside the IFU (p=0.870).^6)^ However, the detailed impact of each factor of IFU was not evaluated in their study. Likewise, Walker et al. reported that overall mortality and aneurysm-related mortality were not affected by IFU adherence in the long-term follow-up. Their Cox proportional hazard model showed that the IFU nonadherence was not predictive for all-cause mortality (HR: 1.0, p=0.910).^10)^

In contrast, several recent studies have focused on the proximal neck angulation associated with adverse events.^4,12–15)^ AbuRahma et al. have revealed its association with perioperative complications, including early type Ia endoleak and reintervention.^13)^ Oliveira et al. reported that severely angulated necks contributed to a higher rate of type Ia endoleaks, and these were significantly associated with freedom from neck-related secondary interventions (86% in the angulated neck group vs. 98% in the control group, p=0.016) in the long term. They also highlighted that cautious enduring follow-up is indispensable in patients treated outside the IFU.^16)^

These opposing perspectives led us to evaluate the threshold of the angulated neck. For this evaluation, we selected the aneurysm sac enlargement evaluated by CT (in the clinical setting), as the indication for reintervention might not be indicated purely for significant sac enlargement and be considered with the patient’s background and comorbidities.

Multivariate analysis examining the anatomical features on CT scan related to IFU revealed that the angle of the proximal LZ (HR: 1.02, p=0.010) and diameter of the proximal LZ (HR: 1.11, p=0.042) were associated with late sac enlargement. Analysis of the angle of the proximal LZ with the ROC analysis showed that setting the cutoff value of the proximal neck angle at 47° indicated a sensitivity of 83% and a specificity of 51% to predict sac growth. A proximal neck angle of 47° was the threshold for sac enlargement after EVAR.

As the predictors for sac enlargement, several studies have reported the neck angle >60° was only predictable, but other degrees have yet to be investigated.^12–17)^ The subgroup comparison in our study, limited to the angulated group, was conducted for the proximal neck angle of 60°, the angle for the IFU. Interestingly, the rates of freedom from sac enlargement indicated no difference between the two groups, and proximal neck angle >47° was again identified as the threshold for developing late sac enlargement even if the AAA was within the IFU.

Among all types of endoleaks after the EVAR, type Ia endoleak was recognized as the failure of EVAR and directly related to sac enlargement. Previous studies have reported the incidences of persistent type Ia endoleaks, ranging from 2.5% to 11.4%, and type Ia endoleaks have reportedly occurred in several stent graft systems.^12–17)^

Regarding the mechanism underlying the persistent type Ia endoleak due to neck angulation, Rahmani et al. reported that proximal angulation decreases the necessary pull-down force and results in dislodging of the endograft in bovine aorta. This in vitro study showed that the pull-down forces decrease in accordance with the neck angle at 0°–90° (Cook Zenith Flex device from 59.8 to 48.9 N, Medtronic Endurant device from 29.9 to 25.8 N, and Medtronic Talent device from 6.0 to 5.5 N).^18)^ From another point of view, De Bock et al. reported using the in vitro test to show that proximal kinking of the device can occur and result in the presence of type Ia endoleak in an angle between the suprarenal aorta and proximal neck above 60°.^14)^

Similar to previous studies,^12–17)^ the current study revealed that the incidence of persistent type Ia endoleak was higher in the angulated group, while its freedom rate for persistent type Ia endoleak was found to be significantly lower (p=0.010). This fact indicated that countermeasures against severe proximal neck angulation during EVAR should be mandatory. As other special technique for proximal neck angulation, snorkel technique has also been reported to prevent the occurrence of type Ia endoleaks.^19, 20)^ In the future, this method may be considered for long-term treatment in addition to current strategies.

In terms of the mechanism of persistent type Ia endoleak due to proximal neck angulation, the pull-down forces by angulation for each device might be considered^18)^ as the different HR of each devices led us to speculate the influence of different trackabilities. However, this speculation should be investigated through a randomized controlled trial (to avoid the selection bias of the device) to ensure the relation between the persistent type Ia endoleak and the specific devices. Additionally, our multivariable analysis indicated the significant effect of reverse-tapered neck for persistent type Ia endoleak.^21)^ Therefore, the additional complex factors including the different trackabilities of a device and reverse-tapered shape of the angle of the proximal LZ were suggested as the mechanism of persistent type Ia endoleak post-EVAR. However, the number of patients having reverse-tapered neck was only 8 (5.0%), and this was not identified as a predictor of sac growth post-EVAR; thus further investigation is needed to resolve this issue.

### Limitations

One of the limitations of our study is having a small sample size for observational retrospective study on a specific cohort who underwent EVAR. Moreover, several data were excluded from the study due to an isolated iliac aneurysm, additional embolization procedure, usage of warfarin, or an insufficient follow-up survey. There was no major device identified as the risk factor for sac enlargement post-EVAR. These results were not presented in this study, because no randomized study was conducted and aneurismal features including proximal neck conditions were not matched. Lastly, the effects of postoperative type II endoleak on sac enlargement could not be completely denied, even if it was statistically not significant.

## Conclusion

The angle of the proximal LZ was found to be an independent risk factor for the development of aneurysm sac growth post-EVAR. The incidence of persistent type Ia endoleaks was found to be significantly higher in the angulated group. Patients with a neck angulation of 47° or more could be considered as the high-risk group for sac growth post-EVAR.
