# Supplementary material for: Preoperative Neck Angulation is Associated with Aneurysm Sac Growth Due to Persistent Type Ia Endoleak after Endovascular Abdominal Aortic Aneurysm Repair
Source: Ann Vasc Dis. 2020 Sep 25;13(3):261–8. doi: 10.3400/avd.oa.20-00057 (PMC7751068; doi:10.3400/avd.oa.20-00057)
Supplement: Supplementary Data [file avd-13-3-oa.20-00057_s001.pdf]

**Table S1** Preoperative CT findings related to IFU and Cox regression analysis: Predictors of persistent type Ia endoleak

| Covariate | | HR | 95% CI | p value | HR | 95% CI | p value |
|---|---|---|---|---|---|---|---|
| | | Uni-variate | | | Multi-variate | | |
| AAA size | | 1.08 | 0.98–1.18 | 0.18 | | | |
| Suprarenal angulation | Angle | 1.03 | 1.00-1.06 | 0.066 | | | |
| Proximal LZ | Length | 0.95 | 0.89–1.02 | 0.18 | | | |
| | Diameter | 1.14 | 0.93-1.40 | 0.19 | | | |
| | Angle | 1.04 | 1.01-1.07 | 0.012 | 1.04 | 1.01-1.07 | 0.012 |
| | Calcification | 1.41 | 0.17–11.4 | 0.75 | | | |
| | Mural thrombus | 4.29 | 0.51-35.9 | 0.18 | | | |
| | Reverse taper | 6.62 | 1.31-33.6 | 0.023 | 6.27 | 1.21-32.4 | 0.029 |
| | Taper | 0.048 | 0.00-4622 | 0.81 | | | |
| Terminal aorta | Diameter | 1.01 | 0.91–1.12 | 0.88 | | | |
| Distal LZ | Length | 1.00 | 0.94-1.06 | 0.87 | | | |
| | Diameter | 1.00 | 0.86-1.15 | 0.92 | | | |
| Access route | Diameter | 1.19 | 0.65-2.20 | 0.57 | | | |
| Embolization of bilateral IIAs | | - | - | - | | | |

AAA: abdominal aortic aneurysm; CI: confidence interval; HR: hazard ratio; IIA: internal iliac artery; LZ: landing zone
